# A pan-cancer analysis of secreted Frizzled-related proteins: re-examining their proposed tumour suppressive function

**DOI:** 10.1038/srep42719

**Published:** 2017-02-20

**Authors:** Krista Marie Vincent, Lynne-Marie Postovit

**Affiliations:** 1 Department of Oncology, Faculty of Medicine and Dentistry, University of Alberta, 114th St and 87th Ave, Edmonton, AB, T6G 2E1, Canada; 2 Department of Anatomy and Cell Biology, Faculty of Medicine and Dentistry, University of Western Ontario, 1151 Richmond St, London, ON, N6A 3K7, Canada

## Abstract

Secreted frizzled-related proteins (SFRPs), containing five family members (SFRPs 1–5) are putative extracellular Wnt inhibitors. Given their abilities to inhibit Wnt signalling, as well as the loss of SFRP1 in many cancers, this family is generally considered to be tumour suppressive. In this study we analyzed gene expression, promoter methylation and survival data from over 8000 tumour and normal samples from 29 cancers in order to map the context-specific associations of SFRPs 1–5 with patient survival, gene silencing and gene expression signatures. We show that only *SFRP1* associates consistently with tumour suppressive functions, and that *SFRP2* and *SFRP4* typically associate with a poor prognosis concomitant with the expression of genes associated with epithelial-to-mesenchymal transition. Moreover, our results indicate that while *SFRP1* is lost in cancer cells via the process of DNA methylation, *SFRP2* and *4* are likely derived from the tumour stroma, and thus tend to increase in tumours as compared to normal tissues. This in-depth analysis highlights the need to study each SFRP as a separate entity and suggests that SFRP2 and SFRP4 should be approached as complex matricellular proteins with functions that extend far beyond their putative Wnt antagonistic ability.

Secreted Frizzled-related proteins (SFRPs) were initially described as tumour suppressor genes when *SFRP1* was found to be downregulated by loss of heterozygosity or promoter methylation in breast and colorectal cancer cell lines^1^,^2^. Given their crucial role in Wnt signalling, and in development, the *SFRP* gene family was quickly recognized for its potential to modulate tumourigenic behaviour.

SFRPs 1–5 are secreted glycoproteins of ~300 amino acids in length, which fold into two independent domains: (1) a N-terminal cysteine-rich domain (CRD), and (2) a C-terminal netrin-like domain (NTR)^3^. The cysteine-rich domain shares considerable sequence homology with Fzd receptors, and due to this molecular mimicry, SFRPs were immediately recognized for their potential to sequester Wnt ligands away from receptor complexes and ultimately antagonize Wnt signalling^4^,^5^. Wnt signalling pathways have been shown to play central roles in cell survival, proliferation, fate determination, polarity, and tissue patterning. Unsurprisingly, dysregulation of Wnt-associated pathways is a key event in the development of many types of cancer. In general, constitutive activation of Wnt signalling (eg. through stabilizing beta-catenin mutations) is recognized to contribute to tumourigenesis^6^. Thus, due to their ability to antagonize Wnt signalling, and their frequent epigenetic silencing, *SFRP*s were initially designated as tumour suppressor genes and many studies have gone on to support this proposed functional role (reviewed in ref. 7).

However, accumulating evidence suggests that they may also promote tumourigenesis in certain contexts. One instance is canine mammary gland tumours, where SFRP2 was found to be overexpressed and induces cancerous transformation in normal mammary epithelial cells. In this case, SFRP2 associated with a fibronectin-integrin extracellular matrix protein complex, and this interaction mediated cell adhesion and blocked apoptosis^8^,^9^,^10^. In metastatic renal cell carcinoma, SFRP1 was found to be upregulated, concomitant with a hypomethylated promoter region. Functionally, knocking down SFRP1 resulted in increased apoptosis and decreased invasive potential^11^. Furthermore, in renal cancer, SFRP2 was also shown to have oncogenic potential; SFRP2 promoted both *in vitro* cellular proliferation and *in vivo* tumour growth^12^. Deciphering the complex effects of SFRPs on tumour progression is likely complicated by the local context of Wnt signalling components, differences between SFRP family members and the unknown impact of NTR domain interactions.

Recent transcriptional and genomic profiling of thousands of patient tumour samples by The Cancer Genome Atlas (TCGA) has enabled a thorough investigation of the functions of the SFRP gene family across different types of cancers. In this study, we investigate the context-specific associations of *SFRP1*–*5* expression in over 8000 tumour and normal samples from 29 different cancers. We show that the putative tumour suppressor function is not consistent between members of the *SFRP* family and that specific *SFRP*s behave in a cancer type-dependent manner. We found that *SFRP1* is the only family member whose expression is consistently decreased in primary cancer samples as compared to associated normal tissues. Moreover, this loss of *SFRP1* expression correlates with gene promoter methylation. Despite these abstruse associations, *SFRP2* and *4* expression consistently clusters together, suggesting a common gene program. We found that *SFRP2* and *4* expression is tightly correlated to stromal content, and forms part of a common epithelial-to-mesenchymal transition (EMT) gene program that is expressed in a multitude of different cancers.

## Results

### Association of *SFRP*s with patient survival reveals strong correlations, but inconsistency between family members and cancer type

**Figure 1:**
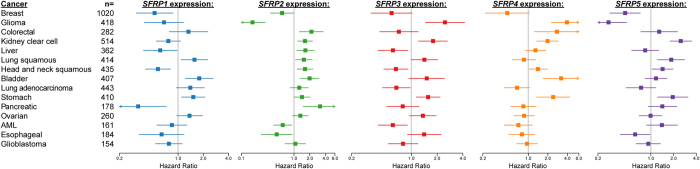
Association of *SFRP* expression with patient survival across different cancer types. Hazard ratios and 95% confidence intervals for overall survival, by cancer type. The forest plot shows the overall survival advantage or disadvantage of increased *SFRP* expression (high *versus* low as stratified by ROC curve) by cancer type, unadjusted for other covariates. The vertical line represents a hazard ratio of one, where there are no survival differences between the two groups.

We were first interested in determining if *SFRP* expression was associated with favourable patient outcomes, as suggested by their proposed tumour suppressor function. We looked at primary tumours from fifteen different cancer types (Supplementary Fig. 1) and dichotimized *SFRP* expression into high and low expressors by ROC curve. Univariate Cox regression analysis was conducted and Kaplan-Meier curves were constructed based on overall survival (Fig. 1, Supplementary Fig. 2). To determine the robustness of these associations, re-sampling analysis was conducted (Supplementary Fig. 3). In general, we found that S*FRP* expression was frequently significantly associated with patient outcomes. However, the direction of that association varied with regards to the particular SFRP isoform queried and the cancer type. Despite this, we observed select consistent cancer-specific or gene-specific effects: For example, high expression of any *SFRP* was associated with poor prognosis in stomach cancer; and high expression of SF*RP4* only associated with poor outcomes (p < 0.05). In colorectal cancer, where promoter *SFRP* methylation and functional studies in cell lines have implicated their role as tumour suppressive^13^,^14^, we found that high expression of *SFRP2* and *SFRP4* is associated with poor patient outcomes (*SFRP2*: HR = 2.14 [1.27–3.58], p = 0.004; *SFRP4*: HR = 2.76 [1.25–6.08], p = 0.01).

### Expression of *SFRP1* and *5*, but not other *SFRP*s, is lost in primary tumours

**Figure 2:**
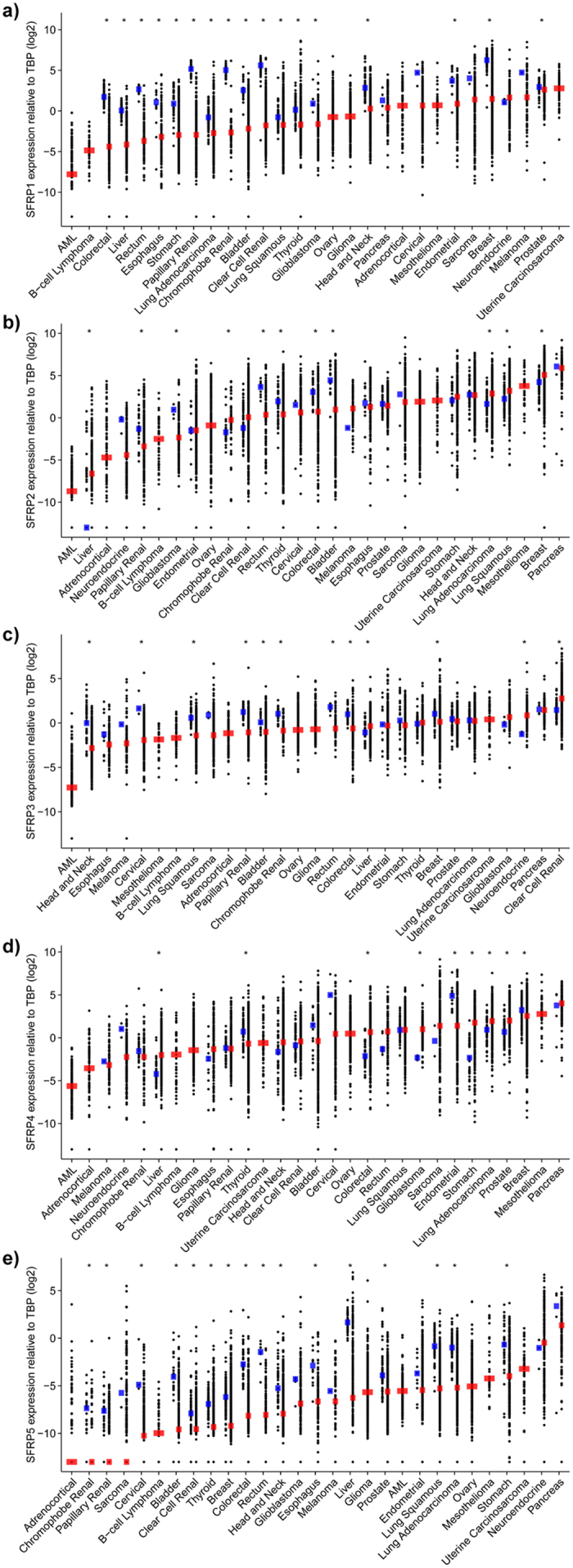
Expression levels of *SFRP*s in normal and cancerous tissue types. Expression levels of (**a**) *SFRP1*, (**b**) *SFRP2*, (**c**) *SFRP3*, (**d**) *SFRP4*, and (**e**) *SFRP5* in patient samples. Each data point represents the *SFRP* expression levels (log_2_[RSEM normalized values relative to TBP]) of one tumour or normal sample. Horizontal bars indicate median expression values for normal (blue) or primary tumour (red) samples. Zero value *SFRP* expressors are plotted at the bottom of the y axis. Comparisons between the normal and tumour expression values were performed using the Mann-Whitney U test to determine significance (*p < 0.05).

One of the defining features of *SFRP* expression during tumourigenesis is a decrease in gene expression. While much of this work has been conducted on normal and cancer cell lines, we investigated the expression levels of all five genes in over 8,000 primary tumours, and 780 associated normal tissues from 29 different cancers (Fig. 2, Supplementary Tables 1 and 2). *SFRP1* and *5* consistently have lower expression in primary tumour tissue compared to normal tissue. However, expression of *SFRP2, SFRP3* and *SFRP4* were often unchanged or even increased in tumour tissue, indicating that they do not undergo the same silencing process as *SFRP1* and *5*.

**Figure 3:**
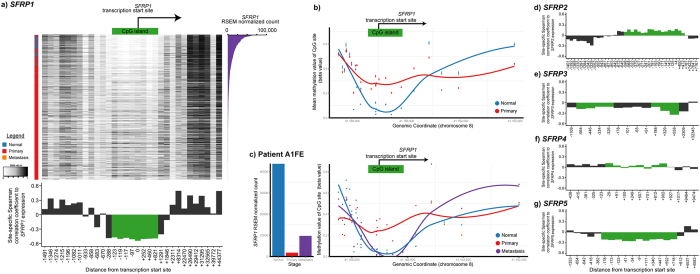
*SFRP1* promoter methylation correlates to *SFRP1* gene expression in breast cancer. (**a**) Heatmap depiction of *SFRP1* methylation levels of individual CpG sites (beta values: black, methylated; white, unmethylated) in breast tumour samples ordered from high *SFRP1* expression levels (*top*) to low *SFRP1* expression levels (*bottom*). Left bar indicates sample source: blue, normal samples; red, primary sample; orange, metastatic sample. Right barplot depicts *SFRP1* expression levels (RSEM normalized values). Bottom barplot depicts Spearman’s correlation coefficients for site-specific methylation levels (beta values) to *SFRP1* RSEM normalized values. (**b**) Scatterplot depicting average CpG methylation values +/− SEM of the *SFRP1* genomic locus of normal (blue) and primary (red) breast cancer tumour samples. Curves represent loess smoothed data. (**c**) Barplot of *SFRP1* expression levels (RSEM normalized values) in normal, primary tumour and metastatic tumour samples from the same patient. Scatterplot of CpG methylation values of the *SFRP1* genomic locus in normal, primary and metastatic tumour samples from the same patient. Barplot of Spearman’s correlation coefficients for site-specific methylation beta levels to (**d**) *SFRP2*, (**e**) *SFRP3*, (**f**) *SFRP4*, and (**g**) *SFRP5* RSEM normalized values.

Regulation of *SFRP* gene expression is commonly observed at the epigenetic level, through DNA methylation, but has also been observed at the genetic level, through loss of heterozygosity^1^,^2^,^15^,^16^. We investigated these possibilities in breast cancer by looking at how copy number events and CpG site methylation correlates with *SFRP* expression levels. We found that there were no copy number alterations that consistently associated with gene levels (Supplementary Fig. 4). At the epigenetic level, methylation of the *SFRP1* promoter region was strongly inversely correlated with *SFRP1* gene expression (Fig. 3a). Methylation of this region in primary tumours was also observed to increase as compared to matched normal tissues at both a cancer-specific and a patient-specific level (Fig. 3b,c). Gene expression did not correlate with CpG methylation for any other *SFRP*s, indicating that their expression is regulated at a different level (Fig. 3d–g).

### SFRP2 and 4 expression in tumours is likely contributed by stroma

**Figure 4:**
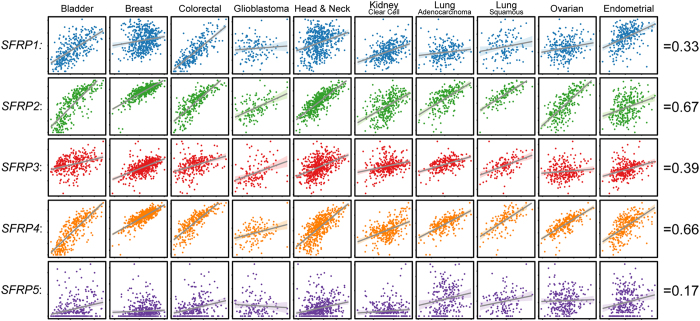
Correlation of *SFRP* expression values to Stromal Scores in various cancers. Scatterplot representations of *SFRP* RSEM expression values (y axes) to Stromal Scores (x axes, as determined by the ESTIMATE algorithm) in bladder cancer, breast cancer, colorectal cancer, glioblastoma, head and neck cancer, clear cell kidney cancer, lung adenocarcinoma, lung squamous cell carcinoma, ovarian cancer and endometrial cancer. Lines indicate linear regression lines; shading indicates confidence interval. Values (right) indicate average Spearman’s correlation coefficients of the individual *SFRP* family members in the described cancers.

A possible alternative mechanism governing alterations in SFRP expression in tumours is that SFRPs are differentially expressed by various cell types in the tumour microenvironment. Several studies have indicated that SFRPs may be expressed by tissue stroma^17^,^18^,^19^ and we investigated that possibility by correlating *SFRP* expression to tumour-specific Stromal Scores (as determined by the ESTIMATE algorithm, Fig. 4). We found that *SFRP2* and *SFRP4* strongly correlated with Stromal Scores in the fourteen cancer types investigated, with an average Spearman’s correlation coefficient of 0.67 and 0.66, respectively. Furthermore, single cell RNA-sequencing in breast cancer suggests that *SFRP2* and *SFRP4* expression is restricted to cells identified as stromal (Supplementary Fig. 5). By contrast, expression of *SFRP1, 3*, and *5* only weakly or conditionally associate with Stromal Scores: for example, *SFRP1* expression strongly correlates with Stromal Scores in colorectal cancer, a cancer where *SFRP1* promoter methylation has been demonstrated to occur in both cell lines and patient samples^13^,^14^.

### Common pan-cancer gene program associated with *SFRP2* and *4* expression

**Figure 5:**
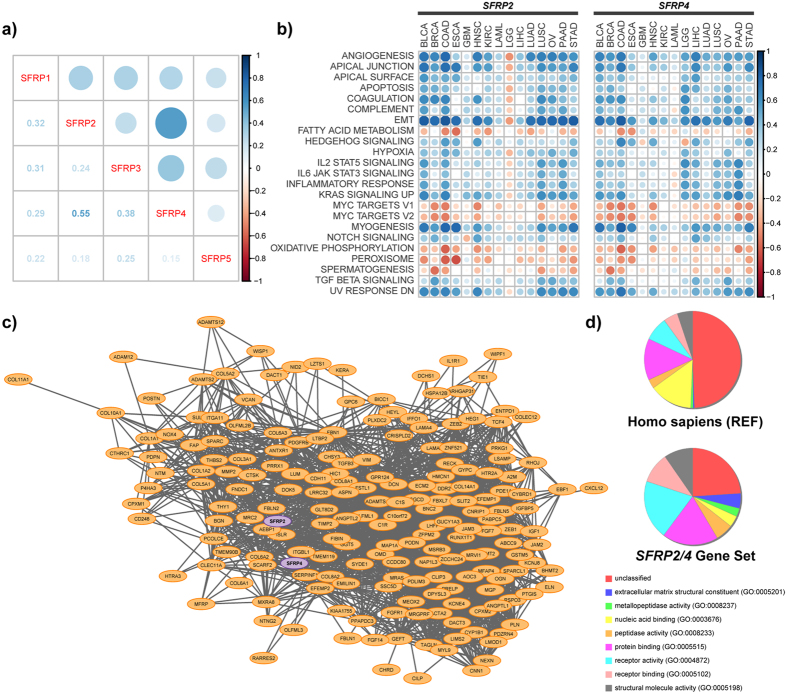
*SFRP2* and *SFRP4* expression strongly associates with an EMT signature. (**a**) Correlation matrix of the expression levels of different *SFRP* family members (log_2_[RSEM normalized values + 1]) in primary tumours. Average of Pearson’s correlation coefficient of gene-gene pairs from 15 different cancers. (**b**) Correlation matrix of *SFRP2* and *SFRP4* expression values (log_2_[RSEM normalized values + 1]) with individual gene sets (GAGE determined enrichment values for MSigDb Hallmark gene sets) in 15 different cancers. Gene sets were included if they had an absolute correlation coefficient of over 0.5 with *SFRP2* or *4* expression values. (**c**) Correlation network analysis of *SFRP2* and *4* reveals a common gene set. The network displayed genes whose expression strongly correlates (r > 0.5) with *SFRP2* and *4* in over 50% of cancers where they are a poor prognostic factor. Nodes colours indicate *SFRP* member location (purple, *SFRP*s; orange, others); edges represent average correlation coefficient >0.8 in the STAD dataset. (**d**) *SFRP2* and *4* correlated genes (n = 180) were classified using PANTHER Classification System based on molecular function.

When expression levels of the various *SFRP*s were correlated to each other (Supplementary Fig. 6) and averaged across cancers, we found *SFRP2* and 4 expression to be tightly correlated (Fig. 5a). This suggests that those two *SFRP*s share a common gene program. Furthermore, the pattern of correlation of *SFRP2* and *4* expression to gene set enrichments are highly concordant, with tight correlations to EMT and angiogenesis gene sets (Supplementary Fig. 7, Fig. 5b). Correlation network analysis of *SFRP2* and *4* reveals a common gene program of 180 genes that are tightly correlated (r > 0.5) across multiple cancers (Fig. 5c). This program includes previously identified key EMT proteins such as ZEB1, ZEB2, VIM, and MMP2. Gene ontology reveals an enrichment of extracellular matrix constituents and metallopeptidase activity ontologies in the identified *SFRP2/4* gene set (Fig. 5D). This correlation network analysis and gene ontology strengthens the notion that SFRP2 and 4 have stromal functions — distinct from their proposed Wnt antagonistic activity, and that they may promote processes such as cellular invasion and metastasis.

## Discussion

This is the first study of its kind to systematically investigate the survival associations and context-specific interactions of secreted Frizzled-related protein family members. Our results contradict the notion that SFRPs are tumour suppressive across all cancers. Indeed, in many cancers, high expression is associated with poor patient outcomes. We show that while *SFRP1* is frequently silenced in many cancers through methylation, the other *SFRP*s do not undergo this silencing event. In fact, *SFRP2* and *SFRP4* expression is often increased in tumours and are tightly correlated to Stromal Scores, suggesting that their expression is produced by the tumour stroma. Gene ontology strengths this observation, with both genes contributing to a common pan-cancer gene set that is tightly associated with EMT. Based on these discoveries, we anticipate that SFRP2 and SFRP4 function as complex matricellular proteins, and that they may have functions that extend far beyond the regulation of Wnt signalling.

*SFRP1* was the first of the gene family found to be altered during tumourigenesis. It was discovered in this manner when it was found to be silenced by methylation or loss of heterozygosity in colorectal and breast cancers^1^,^2^. Following these initial reports, others went on to show similar trends and to tie the effects of *SFRP1* loss to increased Wnt-related signalling in a variety of other cancers. We further validated the essence of these studies with our pancancer analysis by showing that in 17 different cancers, SFRP1 expression is significantly downregulated in tumourous tissues compared to normal counterparts. In addition, we confirmed that in breast cancer the downregulation of *SFRP1* associated with promoter methylation. Several groups have proposed the use of *SFRP1* promoter methylation as a cancer biomarker, and our study provides further support for this concept^20^,^21^,^22^,^23^,^24^.

However, our investigation into the other *SFRP*s did not lead to the same conclusions. We found that high expression of *SFRP2* and *4* most often was associated with poor patient prognoses. Furthermore, expression of *SFRP2* and *4* often increased in primary tumours compared to their normal counterparts, and this expression tightly correlated to tumour stromal content. Given that SFRPs are secreted proteins, expression from any cell type has the potential to modulate the tumour microenvironment, and affect tumourigenesis. Since many of the initial studies into SFRP2 and 4 were done using cancer cell lines, which are composed solely of tumour cells, this nuance has likely been largely overlooked. The effects of stromal-derived SFRPs could be completely different from tumour cell-derived SFRPs. For example, only tumour cell-derived, not stromal-derived, MMP-2 and MMP-13 correlate with poor patient outcomes and aggressive tumour phenotypes in ovarian or breast cancer, respectively^25^,^26^. This is in line with multiple studies that are beginning to appreciate and characterize the complexity involved in biomarker generation^27^,^28^,^29^. Moreover, the effects of SFRPs on three-dimensional tumours composed of a plethora of cell types is likely entirely distinct from their effects on cancer cell lines. This theory is strengthened by a recent study of SFRP2 in melanoma^30^. Kaur *et al*. found that SFRP2 expressed by aged fibroblasts drove melanoma angiogenesis and metastasis. This study was unique in that much of the work was conducted in models that incorporated alternative cells types, such as skin reconstructions or transfer of conditioned media. We suggest that future studies investigating the role of SFRP2 or 4 in tumourigenesis consider possible contribution by the tumour microenvironment and incorporate this into model choice.

Despite production by tumour stroma, *SFRP2* or *4* promoter gene methylation may still show promise as a cancer biomarker. Kalmar *et al*. found that despite an increase in *SFRP2* expression in colorectal cancer compared to associated normals, the SFRP2 promoter region became hypermethylated in the cancerous tissues^31^. They used laser-capture microdissection of colonic epithelial cells to show that this increase was not due to expression within the tumour cells themselves. Moreover, the tumour cells had hypermethylation in the *SFRP2* promoter region. This has also been observed in various cell lines, where the *SFRP2* promoter region of normal cell line derivatives are unmethylated, but that region is hypermethylated in tumour cell lines^16^,^32^,^33^. Therefore, despite the stromal contribution of SFRP2 and/or SFRP4, hypermethylation within the tumour cell compartment may still show utility as a clinical biomarker. Given that the robustness of cancer biomarkers is often an issue, the utilization of SFRPs with other biomarkers (individual or signature-based) would likely improve accuracy.

In all solid cancers investigated, *SFRP2* and *4* showed high concordance in terms of associations with survival, correlation to one another and association with enriched gene sets. The exception to this was brain cancers, where these two proteins appear to act independently of one another. In glioma, high *SFRP4* expression is strongly associated with poor outcomes (HR = 3.93 [2.27–6.81], p < 0.0001), while high *SFRP2* expression is strongly associated with favourable outcomes (HR = 0.16 [0.09–0.28], p < 0.0001). In addition, in glioma, *SFRP4* is strongly associated with EMT and angiogenesis gene sets, like in all other cancers. On the other hand, *SFRP2* in glioma is an exception: It is the only cancer where *SFRP2* expression negatively correlates with EMT and angiogenesis gene sets. This disparity may be driven by tissue of origin effects — *SFRP2* has been shown to be highly expressed in the developing neural system whereas *SFRP4* is not expressed in those structures^34^,^35^,^36^. Further studies are needed to clarify the details of this divergence. However, it may provide a unique opportunity to elucidate some of the distinct molecular mechanisms of these two proteins.

## Conclusions

This study is the first of its kind to systematically analyze the *SFRP* gene family in multiple cancers. We focused on gene expression and patient survival data and sought to identify if all *SFRP*s are associated with tumour suppression. We determined that *SFRP1*, the prototypical family member, is downregulated during tumour formation, silenced by methylation and likely follows much of the dogma surrounding this gene family. On the other hand, *SFRP2* and *4* expression levels often increase during tumourigenesis, likely as a result of increased production by the tumour stroma. These two *SFRP*s, which are often associated with poor patient outcomes, form part of a common gene program that is expressed across many cancers and is associated with EMT. We anticipate that as more studies are conducted, new functions will be discovered for these complex matricellular proteins that extend far beyond their putative Wnt antagonistic ability.

## Methods

### Datasets

RNA-seq expression, patient clinical and methylation data were retrieved from The Cancer Genome Atlas Data Matrix on 17 August 2014. For gene expression analysis of normals and primaries, RNASeqV2 (HiSeq) data was used for ACC, BLCA, BRCA, CESC, COAD, DLBC, GBM, HNSC, KICH, KIRC, KIRP, LAML, LGG, LIHC, LUAD, LUSC, MESO, OV, PAAD, PCPG, PRAD, READ, SARC, SKCM, THCA, and UCS. RNASeqV2 (GA) data was used for UCEC. RNASeq (HiSeq) level data was used for ESCA and STAD cancers. *SFRP* expression values (RSEM or RPKM) were normalized to the expression of *TBP* for each tumour sample. Comparisons between the normal and tumour RNA-seq-derived expression values were performed using the Mann-Whitney U test to determine significance. Breast cancer (BRCA) *SFRP* copy number status was accessed using the cBioPortal on 17 May 2015 (http://www.cbioportal.org/). Single cell RNA-sequencing data was accessed on 7 December 2015 for 51 breast cancer cells from tumour BC02 profiled by the Gene Expression Omnibus (GEO) series, GSE75688.

### Survival Analysis

To include the most recently released patient samples, additional RNASeqV2 (HiSeq) and clinical data were downloaded on 20 August 2015 for COAD, LIHC, BLCA, STAD, and ESCA cancers. These data were used in downstream survival analyses. *SFRP* expression values (RSEM normalized values) were dichotomized by receiver operating characteristics (ROC) curves and the Youden index J method was used to determine the optimal cutoff. Survival curves for overall survival (OS) were constructed using the Kaplan-Meier method and significance was determined by log-rank test. The associations between *SFRP* expression (high versus low) and OS were tested in univariate Cox regression models. Re-sampling Cox regression analysis was conducted by calculating hazard ratios on SFRP expression on 70% of the dataset and re-sampled 500 times.

### Methylation analysis

Breast cancer sample associations between individual CpG site methylation (beta values) and *SFRP* expression (log_2_[RSEM + 1]) were calculated using Pearson’s correlation in normal, primary and metastatic samples.

### Tumour purity

Stromal Scores were defined for tumours through the use of the ESTIMATE (Estimation of STromal and Immune cells in MAlignant Tumour tissues using Expression data; original publication^37^) algorithm using RNASeqV2 data or accessed through the MD Anderson Bioinformatics Portal on 15 May 2015 (http://bioinformatics.mdanderson.org/estimate/). Spearman’s correlation coefficient was used to calculate the association of specific genes to Stromal Scores.

### Gene set enrichment analysis

Enrichment for *SFRP* associated gene sets was conducted using Generally Applicable Gene-set Enrichment (GAGE, v2.12.3). Hallmark gene sets were downloaded from the Molecular Signatures Database (http://software.broadinstitute.org/gsea/msigdb) v5.0 on 10 August 2015^38^. Enrichment was calculated against a formulated sample composed of the mean expression values for each gene and sample-specific test statistics were correlated to *SFRP* expression values (log_2_[RSEM + 1]) using Spearman’s rank correlation on a cancer-specific level.

### Correlation network analysis

Correlation network analysis was conducted to determine the context in which *SFRP*s are expressed. Pearson’s correlation coefficient were determined for *SFRP*s (log_2_[RSEM + 1]) to all genes. Correlations were determined for cancers where *SFRP2* is associated with poor prognosis (BLCA, COAD, HNSC, KIRC, LIHC, LUSC, PAAD, and STAD) and for cancers where *SFRP4* is associated with poor prognosis (BLCA, COAD, HNSC, KIRC, LGG, and STAD). Genes were included in the network if their expression values (log_2_[RSEM + 1]) were strongly correlated (Pearson’s correlation coefficient >0.5) in at least 50% of the cancers analyzed for correlation with *SFRP2* and 50% of the cancers analyzed for correlation with *SFRP4*. Cytoscape (v3.2.1) was used to visualize the constructed network^39^. Edges depict an average gene-gene expression correlation coefficient of >0.8 in the STAD dataset.

### Gene ontology

The identified *SFRP2/4* gene set (n = 180) was classified using the PANTHER Classification System (http://www.pantherdb.org, version 10.0, released 2015-05-15)^40^. The genes were classified based on their molecular function and a Statistical Overrepresentation Test was performed on these genes to examine enrichment of GO terms.

### Statistical analysis

We conducted all analyses and visualizations in the RStudio programming environment (v0.98.501). R/Bioconductor packages ggplot2, corrplot, plyr, gplots, matrixStats, survival, and GAGE were used where appropriate.

## Additional Information

**How to cite this article**: Vincent, K. M. and Postovit, L.-M. A pan-cancer analysis of secreted Frizzled-related proteins: re-examining their proposed tumour suppressive function. *Sci. Rep.* **7**, 42719; doi: 10.1038/srep42719 (2017).

**Publisher's note:** Springer Nature remains neutral with regard to jurisdictional claims in published maps and institutional affiliations.

## Supplementary Material

Supplementary Figures and Tables
