# Supplementary material for: A pan-cancer analysis of secreted Frizzled-related proteins: re-examining their proposed tumour suppressive function
Source: Sci Rep. 2017 Feb 20;7:42719. doi: 10.1038/srep42719 (PMC5316967; doi:10.1038/srep42719)
Supplement: Supplementary Figures and Tables [file srep42719-s1.pdf]

A pan-cancer analysis of secreted Frizzled-related proteins: re-examining their proposed tumour suppressive function

Krista Marie Vincent[1,2], and Lynne-Marie Postovit[1,*]

[1]Department of Oncology, Faculty of Medicine and Dentistry, University of Alberta, 114th St and 87th Ave, Edmonton, AB, Canada, T6G 2E1
[2]Department of Anatomy and Cell Biology, Faculty of Medicine and Dentistry, University of Western Ontario, 1151 Richmond St, London, ON, Canada, N6A 3K7
*postovit@ualberta.ca

SUPPLEMENTAL INFORMATION

SUPPLEMENTARY FIGURE LEGENDS

Supplementary Figure 1. Summary of patient survival information for 15 different cancers from the TCGA. Kaplan-Meier depiction of overall survival for the various cancer dataset used. Barplot depicts 5 year survival rate +/- 95% confidence interval.

Supplementary Figure 2. Survival analysis of multiple cancers based on *SFRP* expression. Kaplan-Meier depictions of 5 year overall survival stratified by *SFRP* expression (high expression: red, low expression: blue) in fifteen types of cancer. Patients were dichotimized by ROC curve. Significance was determined by log-rank test and associated p values are displayed in the lower right corners of each plot.

Supplementary Figure 3. Re-sampling analysis of the association of *SFRP* expression with patient survival across different cancer types. Hazard ratios for *SFRP* expression on overall survival were conducted on 70% of the dataset and re-sampled 500 times. Hazard ratios of each *SFRP* across different cancer types were plotted on violin plots.

Supplementary Figure 4. Association of *SFRP* expression with copy number status in breast cancer. Boxplot depicts (a) *SFRP1*, (b) *SFRP2*, (c) *SFRP3*, (d) *SFRP4*, and (e) *SFRP5* expression values (log_2[RSEM normalized values + 1]) against putative *SFRP* copy number status (GISTIC) for breast cancer patients. Significance was determined by one-way ANOVA followed by Tukey's HSD. Boxplots sharing the same letter are not significantly different according to Tukey's HSD test ($p > 0.05$).

Supplementary Figure 5. Single cell analysis of *SFRP2* and *SFRP4* expression in breast cancer. Barplot depicts (a) Stromal and (b) Immune Scores of individual cells from tumour BC02. Cells were identified as stromal or immune if they were >1.75 standard deviations above mean levels. (c) *SFRP2* and (d) *SFRP4* expression values (transcripts per million, TPM) were summarized based on cell type. Bars depict mean expression values.

Supplementary Figure 6. Correlation matrix of *SFRP*s to each other in fifteen types of cancer. Correlation matrix of the expression levels of different *SFRP* family members (log_2[RSEM normalized values + 1]) in primary tumours from multiple cancers. Positive correlations are displayed in blue and negative correlations in red. Colour intensity and size of the circle are proportional to the correlation coefficients. Pearson's correlation coefficients are identified in the corresponding lower left corners. Correlations that are crossed out are not significant ($p > 0.05$).

Supplementary Figure 7. Correlation matrix of *SFRP*s to indicated gene sets in fifteen types of cancer. Correlation matrix of the expression levels of different *SFRP* family members (log_2[RSEM normalized values + 1]) in primary tumours to indicated gene set enrichment. Positive correlations are displayed in blue and negative correlations in red. Colour intensity and size of the circle are proportional to the correlation coefficients.

Supplementary Table 1. Summary statistics of *SFRP* expression in primary tumours for the 29 tumour types used in this study.

Supplementary Table 2. Summary statistics of *SFRP* expression in normal tissues for the 29 tumour types used in this study.

Supplemental Figure 1

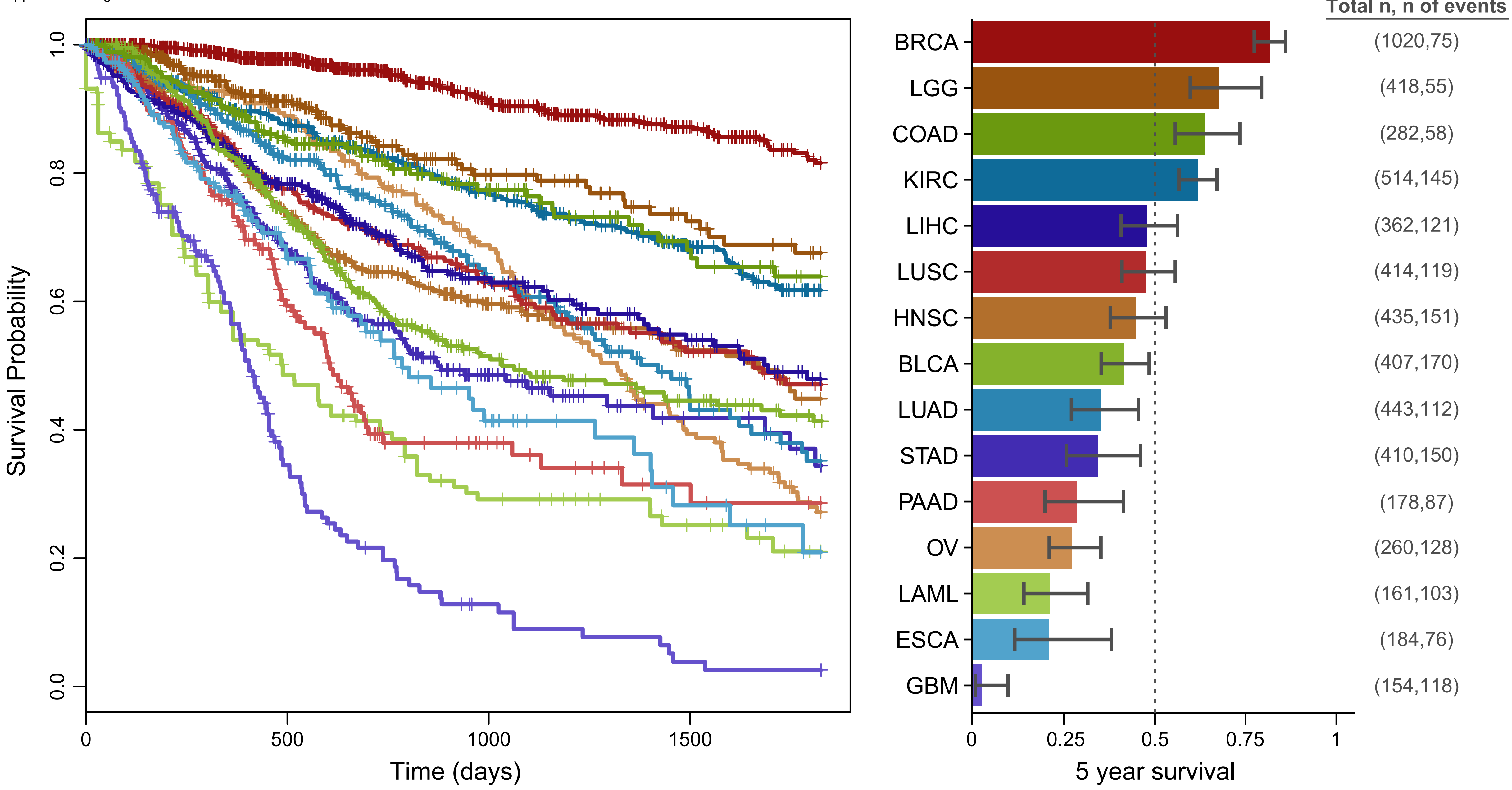

Supplemental Figure 2a

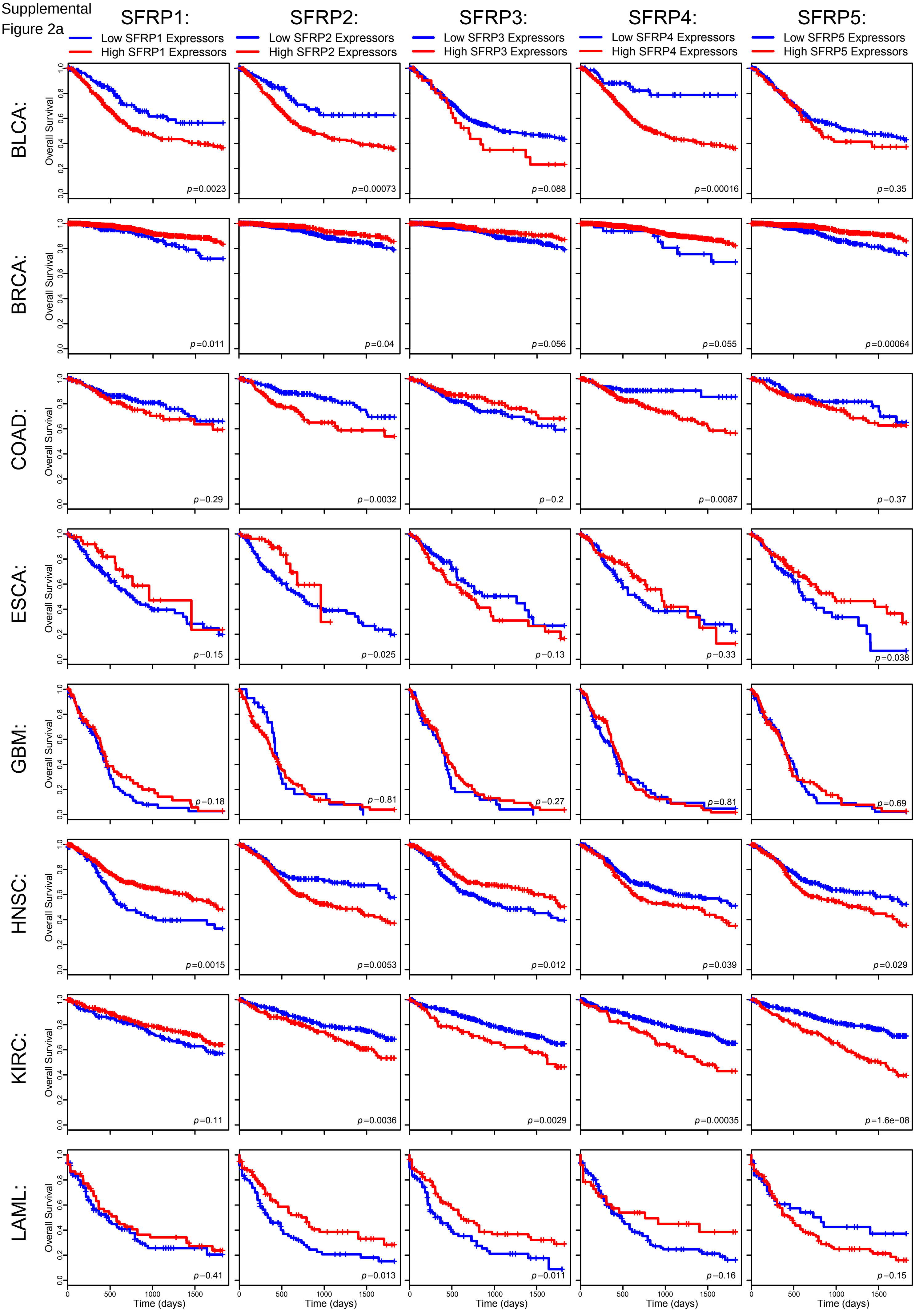

Supplemental Figure 2b

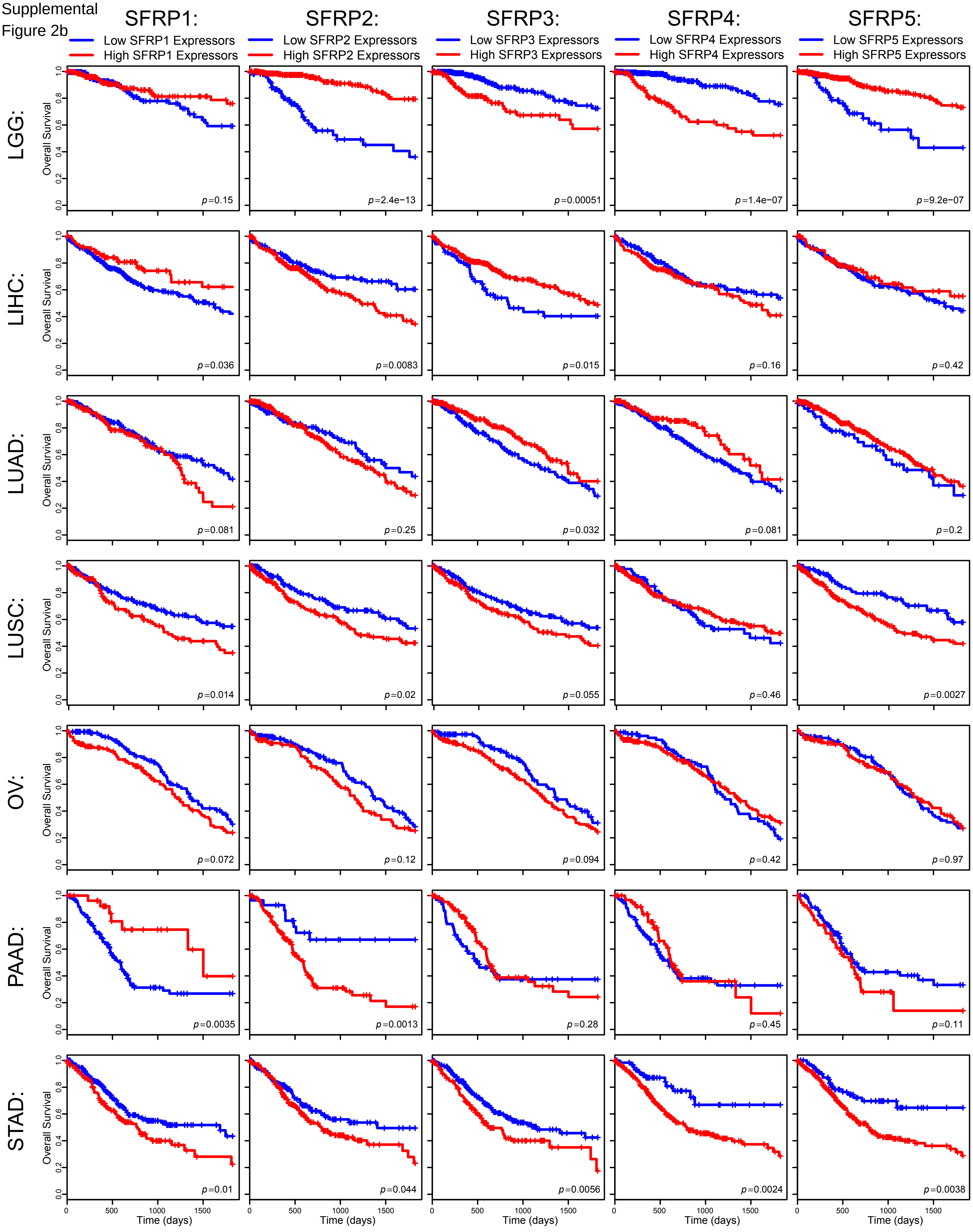

Supplemental Figure 3

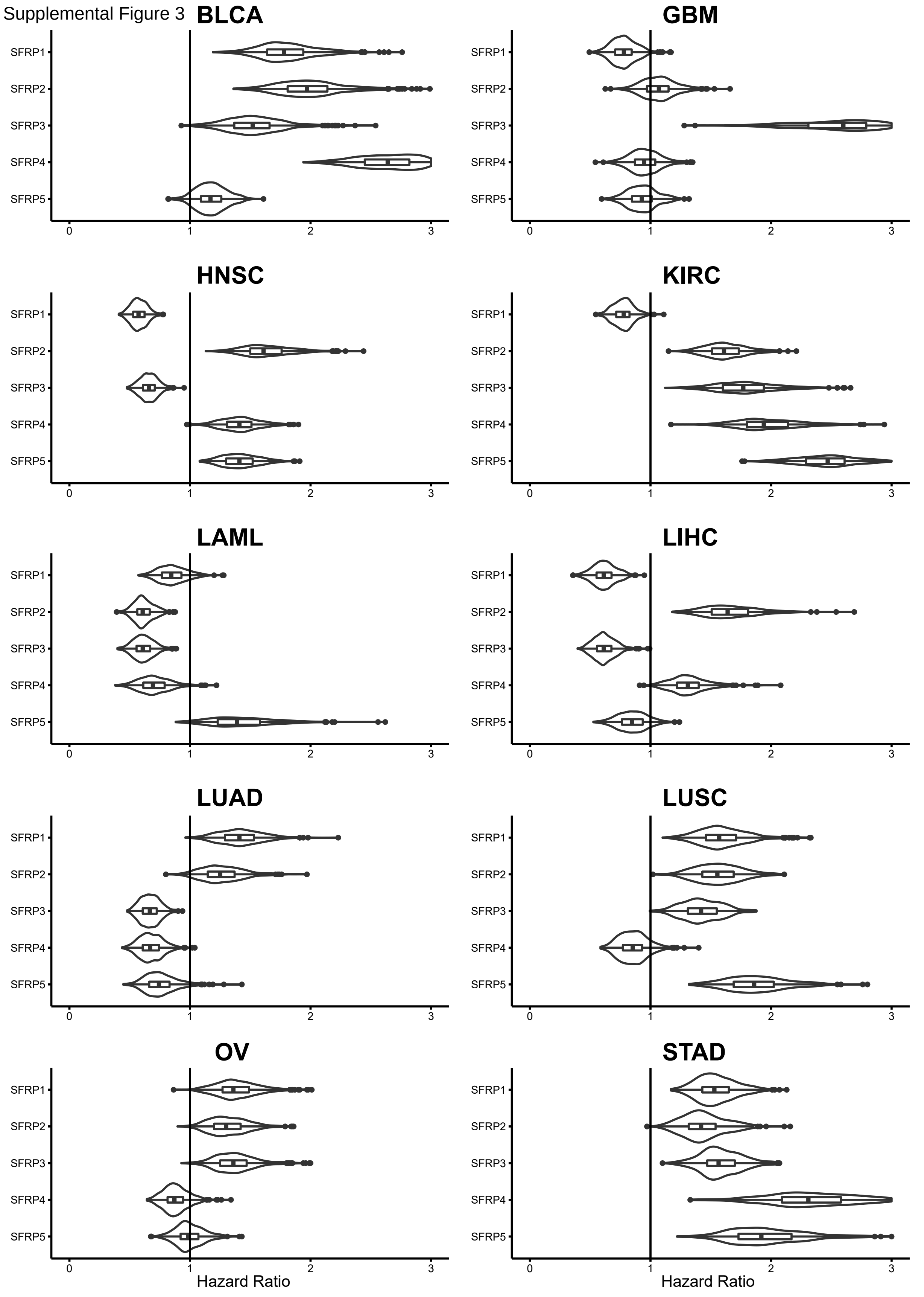

Supplemental Figure 4

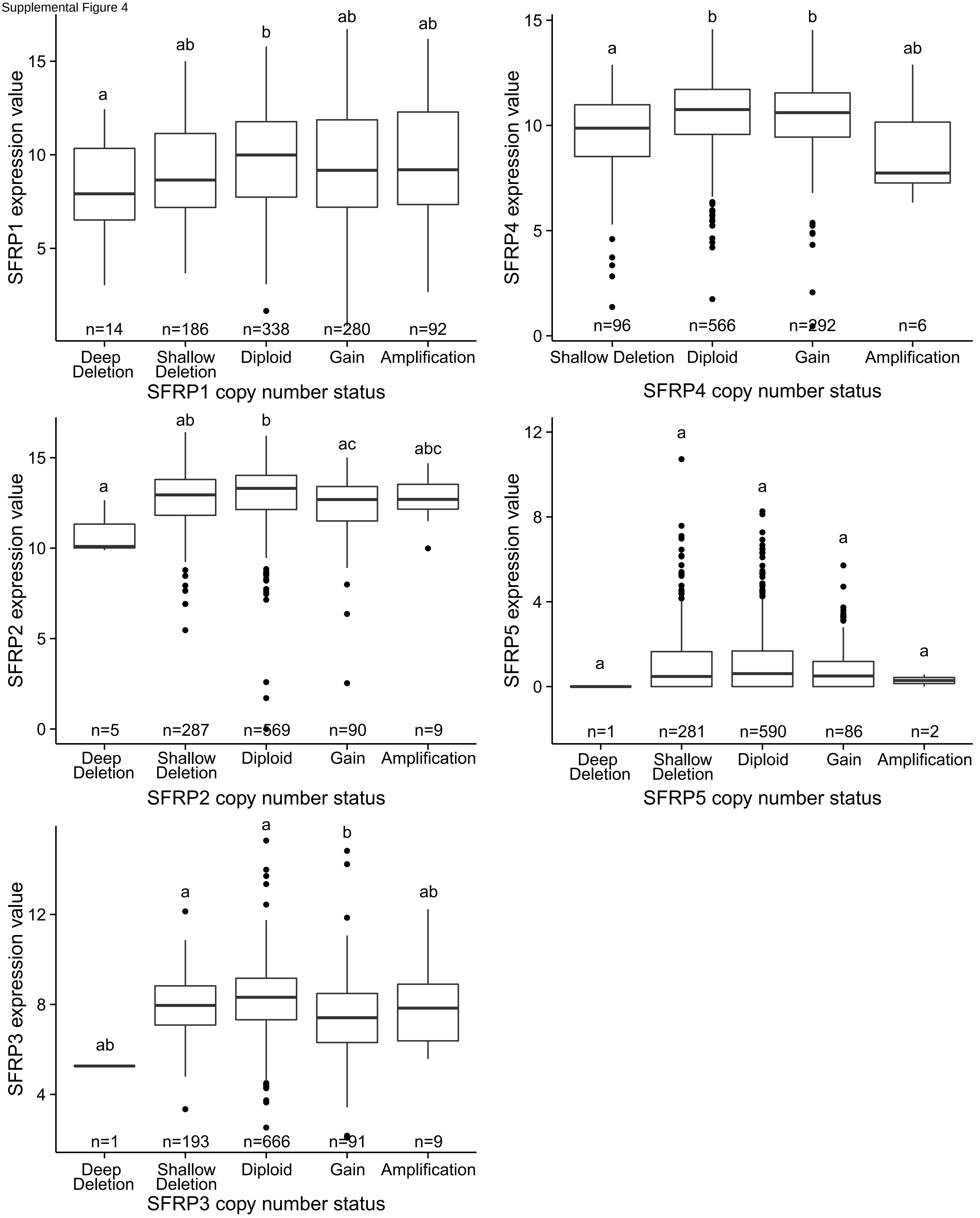

Supplemental Figure 5

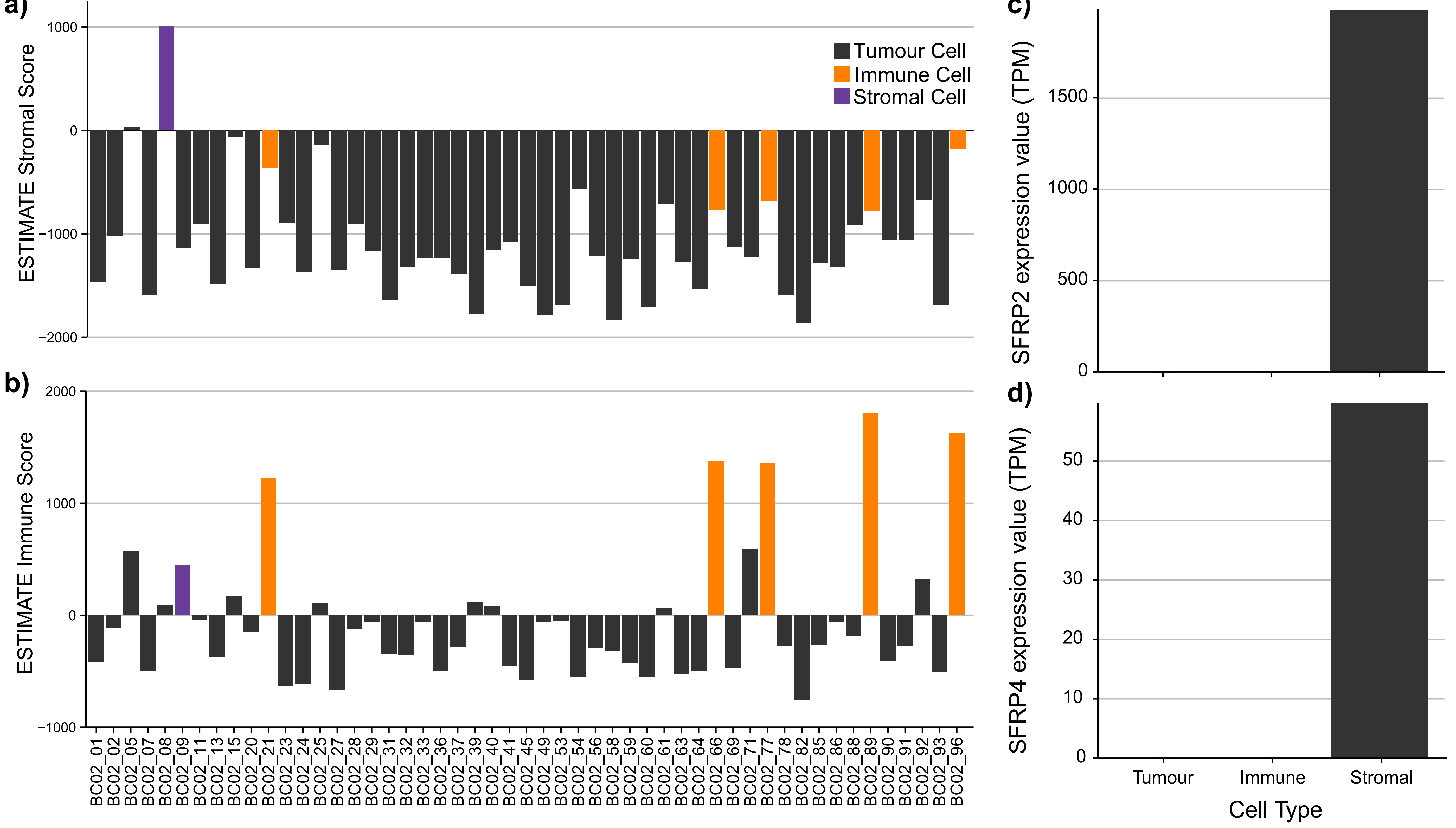

Supplemental Figure 6

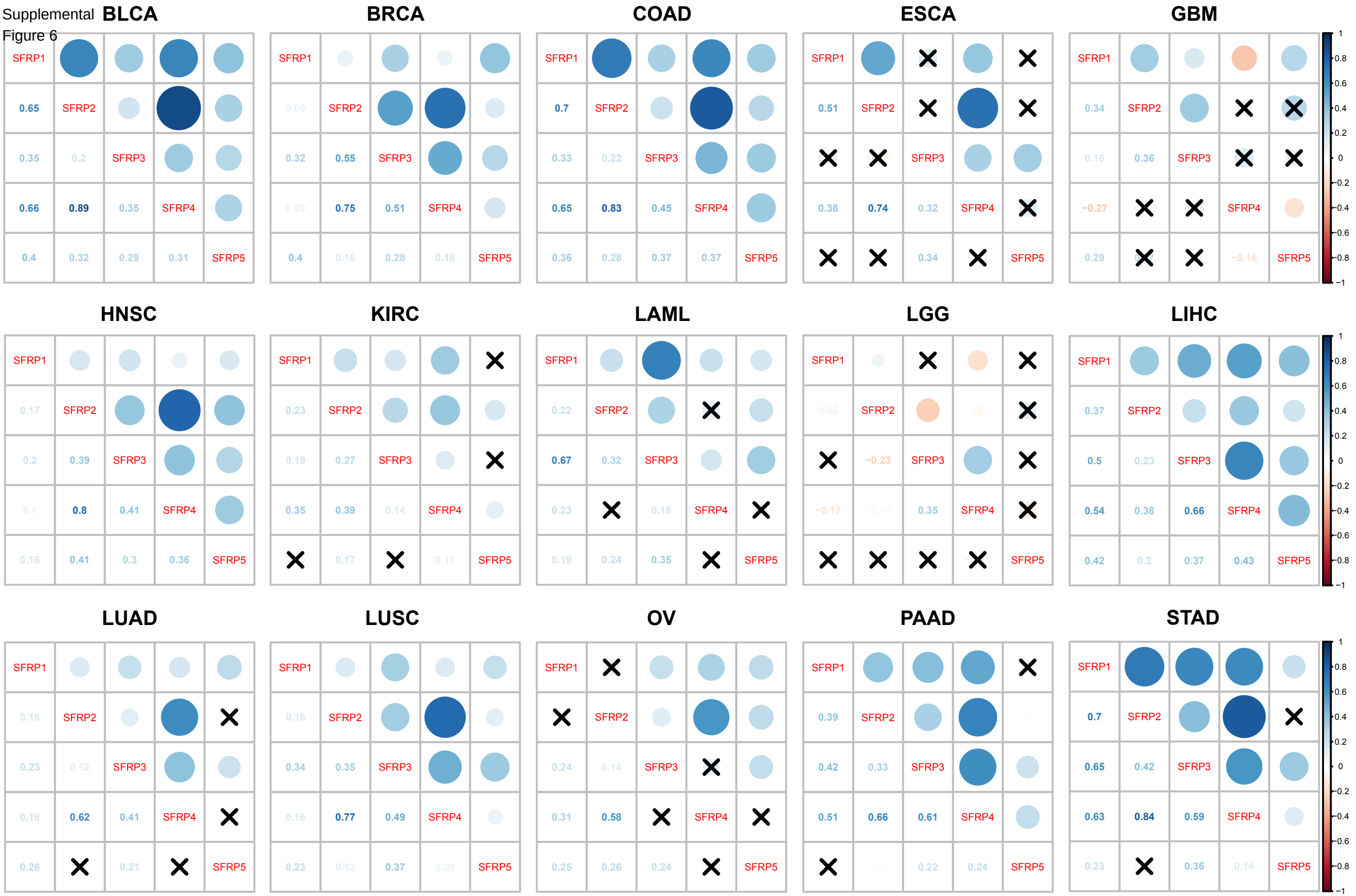

Supplemental Figure 7

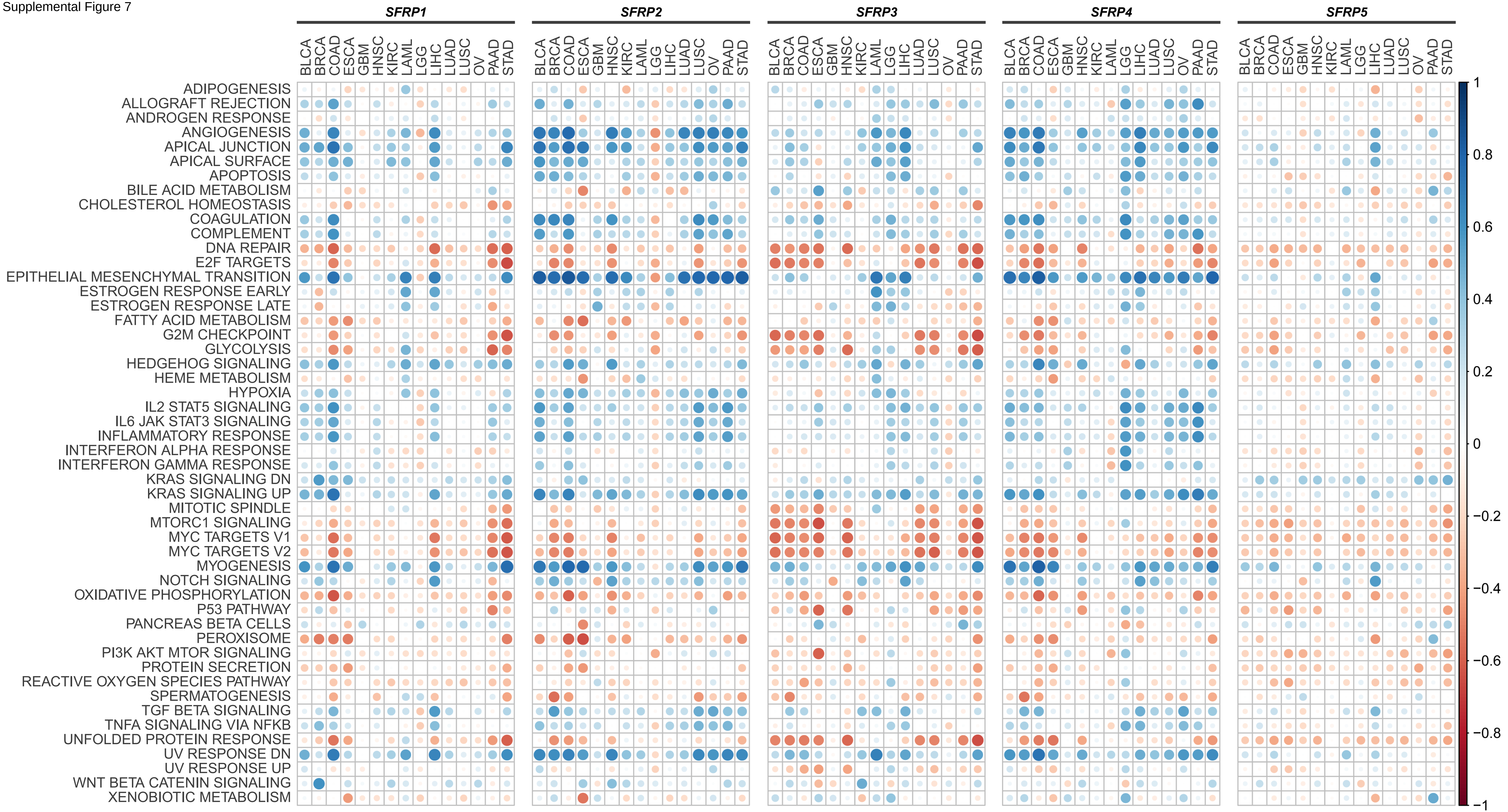

Supplemental Table 1. Summary statistics of SFRP expression in primary tumours for the 29 tumour types used in this study.

			Expression data									
			SFRP1		SFRP2		SFRP3		SFRP4		SFRP5	
Tumour Type	TCGA ID	n	Range	Median	Range	Median	Range	Median	Range	Median	Range	Median
Adrenocortical carcinoma	ACC	79	0.00584-60.90331	1.57944	0-20.195543	0.038278	0.01855-4.84615	0.44737	0-8.96342	0.08537	0-11.77111	0
Bladder urothelial carcinoma	BLCA	310	1-24.83405	0.22133	0-189.10664	1.95221	0.003941-17.4223	0.49892	0-223.84745	0.77636	0-1.153605	0.001302
Breast invasive carcinoma	BRCA	1062	0.0028-398.3573	2.8125	0-370.36	33.58	0.00649-145.1939	1.09666	0.00161-180.4253	5.82701	0-7.110569	0.001685
Cervical squamous cell carcinoma and endoce	CESC	253	0.00077-65.14137	1.59202	0.00195-153.	1.54257	0.00541-50.23005	0.26316	0-155.2068	1.3742	0-0.6299401	0.000821
Colon adenocarcinoma	COAD	273	0-9.60983	0.04798	0-87.2538	1.6535	0.009901-27.7958	0.65581	0.00314-64.3133	1.58837	0-4.607973	0.003533
Lymphoid neoplasm diffuse large B-cell lymph	DLBC	43	0.002635-0.39665	0.0347	0.00056-7.62	0.17682	0.01186-2.38689	0.31162	0-7.5739	0.2585	0-0.0414847	0.000993
Esophageal carcinoma	ESCA	125	0.00141-22.42087	0.10971	0.00074-61.6	2.438	0.01518-4.17078	0.18529	0-18.30090	0.40229	0-1.529435	0.010036
Glioblastoma multiforme	GBM	156	0.00420-70.13783	0.32895	0.00361-22.3	0.19706	0.03043-28.11672	1.59096	0.09075-69.5336	2.00423	0-19.97752	0.008578
Head and neck squamous cell carcinoma	HNSC	497	0.01184-43.86487	1.22222	0.03571-205.	6.375	0.005703-3.72693	0.14067	0.00132-140.501	0.70247	0-4.846954	0.004073
Kidney chromophobe	KICH	66	0.00294-72.24735	0.15947	0.001089-20.	0.839002	0.07941-3.03265	0.55491	0-14.18824	0.2155	0-0.811344	0
Kidney renal clear carcinoma	KIRC	531	0.00432-92.40907	0.29238	0-127.0790	1.0428	0.1317-331.6563	6.7594	0.00319-92.47894	0.75227	0-2.256757	0.001335
Kidney renal papillary cell carcinoma	KIRP	241	0-25.14800	0.13088	0-22.90029	0.09647	0.00885-74.39822	0.48062	0-25.2253	0.4153	0-1.006944	0
Acute myeloid leukemia	LAML	173	0-0.858187	0.00453	0-0.093458	0.002392	0-2.117284	0.00649	0-0.444860	0.02032	0-1.24561	0.02143
Brain lower grade glioma	LGG	513	0.002219-20.74505	0.62651	0.00453-123.	3.73628	0.09541-27.02295	0.61158	0.01057-34.0518	0.36682	0-66.76755	0.01968
Liver hepatocellular carcinoma	LIHC	268	0-8.45124	0.05707	0-11.89032	0.01023	0.0533-36.2656	0.7797	0-47.9025	0.2472	0-120.43881	0.01307
Lung adenocarcinoma	LUAD	488	0-65.75837	0.15199	0.04023-199.	7.27658	0.01982-16.70282	1.17524	0.01346-188.445	3.8599	0-5.396196	0.027429
Lung squamous cell carcinoma	LUSC	491	0.00493-63.42261	0.30612	0.0205-334.7	9.1669	0.009719-9.77187	0.37767	0.01157-57.75582	1.90166	0-7.798091	0.025797
Mesothelioma	MESO	36	0.06606-60.38315	1.62307	0.2933-111.3	13.5762	0.01459-0.96429	0.27614	0.152-164.118	6.874	0.005836-1	0.053438
Ovarian serous cystadenocarcinoma	OV	262	0.00806-105.93238	0.59627	0-88.94866	0.53444	0.02216-37.34842	0.58062	0.00316-97.35991	1.41155	0-14.429923	0.030044
Pancreatic adenocarcinoma	PAAD	124	0.00823-22.29949	1.31975	0.0206-585.9	59.4444	0.2756-38.8793	2.7401	0.04545-95.9166	16.3085	0-40.7250	2.5696
Pheochromocytoma and Paraganglioma	PCPG	179	0.03319-185.48998	3.15909	0-3.40893	0.04715	0.1517-109.6754	1.8048	0.001364-13.1092	0.21298	0-101.4390	0.72137
Prostate adenocarcinoma	PRAD	418	0.002882-28.63309	6.188	0.00609-42.2	2.69929	0.1588-23.4055	1.1456	0.01153-67.65765	4.00541	0-1.528662	0.020559
Rectum adenocarcinoma	READ	91	0.002732-8.786259	0.07849	0.00207-77.5	1.28553	0.07074-10.66667	0.64735	0.00432-60.91293	1.66942	0-0.471074	0.003766
Sarcoma	SARC	212	0.00211-246.92765	2.6637	0-727.7104	3.6773	0.01463-102.4301	0.38471	0.0019-566.4545	2.6268	0-44.7471	0
Skin cutaneous melanoma	SKCM	82	0.0063-356.1323	3.2244	0.06109-22.2	2.14157	0-30.15196	0.20497	0.00365-5.60924	0.11118	0-0.829851	0.01015
Stomach adenocarcinoma	STAD	529	0.1994-14.429726	0.13515	0.00271-254.	5.76262	0.0184-12.3715	0.8426	0.00111-44.4083	3.57855	0-12.93622	0.06636
Thyroid carcinoma	THCA	498	0-395.5872	0.3103	0-227.0570	1.326	0.01075-22.47112	1.03453	0-69.3800	0.625	0-3.590908	0.001555
Uterine corpus endometrial carcinoma	UCEC	370	0.00851-37.36764	1.85028	0-117.78238	0.3563	0.01702-65.94483	0.81482	0.00546-242.2872	2.6518	0-15.753458	0.022858
Uterine carcinosarcoma	UCS	57	0.08728-54.74269	6.88796	0.05937-56.7	4.13761	0.05993-11.55522	1.31512	0.01839-27.57355	0.66182	0-2.06684	0.1076

Supplementary Table 2

Column2	olumn	SFRP1	Column4	SFRP2	Column5	SFRP3	Column6	SFRP4	Column7	SFRP5	Column8
TCGA ID	n	Range	Median	Range	Median	Range	Median	Range	Median	Range	Median
ACC	0										
BLCA	19	0.74-43.28	5.89	.08-108.79	21.78	0.39-2.95	1.06	0.08-12.99	2.76	0-1.49	0.06
BRCA	113	.35-206.11	75.87	1.88-72.70	18.89	0.32-6.22	2.04	0.66-83.83	9.37	0-0.75	0.01
CESC	3	8.38-53.04	26.28	0.02-3.98	2.88	2.05-12.40	3.11	.05-170.54	31.68	0.03-0.93	0.03
COAD	41	0.88-14.11	3.33	1.59-44.80	8.32	0.66-5.99	1.98	0.04-2.06	0.23	0.02-7.75	0.15
DLBC	0										
ESCA	12	.007-10.66	2.18	.04-101.29	3.50	0.05-5.26	0.42	0.003-2.50	0.19	0.01-1.06	0.14
GBM	5	0.90-14.71	1.88	0.93-3.04	1.93	0.53-1.60	0.88	0.16-0.24	0.20	0.007-0.05	0.05
HNSC	43	.98-106.74	7.19	.35-113.94	6.67	0.08-19.97	0.99	0.01-25.55	0.32	0.001-1.58	0.03
KICH	25	0.33-70.50	32.64	0.06-10.51	0.31	0.77-5.94	2.05	0.09-53.59	0.34	0-0.07	0.01
KIRC	72	.08-109.55	49.04	0.04-16.18	0.43	.91-108.99	2.77	0.05-7.95	0.55	0-0.40	0.00
KIRP	32	1.62-75.62	36.02	0.03-9.56	0.40	0.84-5.33	2.36	0.09-2.58	0.44	0-0.08	0.01
LAML	0										
LGG	0										
LIHC	50	0.31-2.94	1.06	0-1.71	0.00	0.14-2.18	0.48	0.008-1.02	0.05	0.85-16.12	3.17
LUAD	58	0.06-3.37	0.58	0.33-26.65	3.12	0.30-4.99	1.23	0.15-14.22	1.91	0.05-4.18	0.51
LUSC	50	0.09-17.10	0.59	0.54-76.30	4.71	0.40-7.49	1.50	0.24-11.49	1.88	0.02-2.98	0.56
MESO	0										
OV	0										
PAAD	4	0.71-7.31	2.76	.05-185.93	82.70	2.03-4.83	3.02	0.47-33.45	18.92	.004-25.90	13.67
PCPG	3	1.91-2.76	2.12	0.27-1.06	0.87	0.41-0.50	0.42	1.03-3.21	2.05	0.11-0.88	0.49
PRAD	52	1.39-33.96	7.79	0.85-13.68	3.15	0.13-4.75	1.36	0.12-14.10	1.61	0.01-8.76	0.07
READ	9	2.24-9.47	6.37	3.95-25.73	12.52	1.77-4.56	3.50	0.26-1.68	0.40	0.04-0.82	0.37
SARC	2	0.07-26.23	18.15	0.52-89.47	44.99	1.47-2.30	1.89	0.23-2.69	1.46	0.007-0.05	0.03
SKCM	1		26.63		0.44		0.90		0.15		0.02
STAD	66	0.05-11.37	1.88	0.02-28.16	4.20	0.06-30.20	1.22	0.005-4.06	0.20	.003-11.50	0.63
THCA	59	0.19-11.15	1.11	0.08-29.21	3.86	0.21-6.53	0.94	0.21-25.76	1.65	0-0.09	0.01
UCEC	11	0.88-47.22	13.28	0.006-3.91	0.35	0.07-10.55	0.89	.64-198.83	29.56	0.006-0.46	0.08
UCS	0										
